# Controlling the Composition and Magnetic Properties of Nano-SrFe_12_O_19_ Powder Synthesized from Oily Cold Mill Sludge by the Citrate Precursor Method

**DOI:** 10.3390/ma12081250

**Published:** 2019-04-16

**Authors:** Bo Liu, Shengen Zhang, Britt-Marie Steenari, Christian Ekberg

**Affiliations:** 1 Institute for Advanced Materials and Technology, University of Science and Technology Beijing, Beijing 100083, China; liubo@ustb.edu.cn; 2 Nuclear Chemistry and Industrial Material Recycling, Department of Chemistry and Chemical Engineering, Chalmers University of Technology, 41296 Gothenburg, Sweden; bms@chalmers.se (B.-M.S.); che@chalmers.se (C.E.)

**Keywords:** SrFe_12_O_19_, nanoparticles, citrate precursor method, industrial waste, magnetic properties

## Abstract

This paper proposes a new method for producing nano-SrFe_12_O_19_ powder by the citrate precursor route using solid waste as a source of iron. This solid iron-containing waste, which exists in the form of an oily sludge, is produced by a cold rolling mill. This sludge was first subjected to a process, including sulfuric acid leaching, oxidation, precipitation, and nitric acid leaching, to obtain an iron nitrate (Fe(NO_3_)_3_) solution. Next, the Fe(NO_3_)_3_ solution was mixed with a strontium nitrate (Sr(NO_3_)_2_) solution obtained by subjecting strontium carbonate to nitric acid leaching. Subsequently, citric acid, as chelating agent, and ammonia water, as precipitating agent, were added to the mixed solution to form a gel. The gel was dried and spontaneously combusted, then annealed at different temperatures for 2 h in flowing air. The effects of the Fe^3+^/Sr^2+^ molar ratio and annealing temperature on the formation, morphology, and magnetic properties of SrFe_12_O_19_ were investigated. The results showed that single-phase SrFe_12_O_19_ powder was obtained by decreasing the Fe^3+^/Sr^2+^ molar ratio from the stoichiometric value of 12 to 11.6 and increasing the annealing temperature to 1000 °C for 2 h. Adjustment of the Fe/Sr molar ratio to 12 and the annealing temperature to 900 °C enabled the magnetic properties to be optimized, including saturation magnetization (Ms) 80.2 emu/g, remanence magnetization (Mr) 39.8 emu/g, and coercive force (Hc) 6318 Oe.

## 1. Introduction

The most important ferrite materials with permanent magnetic properties, M-type ferrites are widely used as magnetic recording media, microwave absorbers, magneto-optics, and other functional materials in practical applications, and they also hold promise for future use in catalysis, biology, and other fields [1,2,3,4]. Among the M-type ferrite materials, SrFe_12_O_19_ does not contain the toxic heavy metal Pb, which contributes considerably to the content of PbFe_12_O_19_. Moreover, the magnetic properties of SrFe_12_O_19_ are slightly superior to those of BaFe_12_O_19_ [5]. Therefore, SrFe_12_O_19_ has received sustained and extensive attention [6,7,8]. Traditionally, SrFe_12_O_19_ is prepared via a solid-state reaction process [9], which mainly involves ball milling of iron and strontium oxides, and subsequent roasting at high temperature (~1200 °C). Although this process is inexpensive and convenient, it is difficult to accurately control the chemical homogeneity, particle size distribution, and crystal defects, thereby resulting in unsatisfactory magnetic properties [10].

Attempts to overcome these problems have led to the development of non-traditional methods, such as co-precipitation [11], sol–gel [12], hydrothermal [13], molten salt-assisted [14,15], and citrate precursor [16,17,18]. Among these methods, the sol–gel and citrate precursor methods enable raw materials to be mixed on the ionic level and subsequent crystallization at low temperature, resulting in the production of uniform nano-SrFe_12_O_19_ [12,19]. Compared with the sol–gel method using metal alkoxide as raw material, the citrate precursor method has a relatively low production cost and simple process. Therefore, the citrate precursor method is considered to be promising for large-scale production of high-performance nano-SrFe_12_O_19_. In recent years, the preparation of nano-SrFe_12_O_19_ by the citrate precursor method has become a popular research topic. Although these studies focused on different aspects, such as process improvement [20,21,22,23] and doping modification [16,18,24], almost all of these studies utilized chemically pure nitrates as starting materials.

The rapid development of modern industry has caused the disposal of industrial solid waste to become a matter of serious global concern. In view of the wide application range and huge annual demand for SrFe_12_O_19_, the production of SrFe_12_O_19_ from industrial solid waste is not only able to realize the large-scale utilization of solid waste, but also to significantly reduce the production cost of SrFe_12_O_19_. Therefore, related studies have aroused widespread interest. Hessien et al. [25] synthesized SrFe_12_O_19_ powder with maximum saturation magnetization (Ms) 74.15 emu/g, remanence magnetization (Mr) 38.95 emu/g, and coercive force (Hc) 3455 Oe, using Egyptian celestine ore as a source of strontium, via a co-precipitation method. Xie et al. [26] reported a method for preparing SrFe_12_O_19_ powder with Ms 52.7 emu/g, Ms 29.6 emg/g, and Hc 3346 Oe from industrial strontium slag by chemical co-precipitation. Oily cold rolling mill (CRM) sludge is a metallurgical by-product produced during the process of cold rolling strip steel. In our previous research [27], SrFe_12_O_19_ powder with Ms 62.6 emu/g, Mr 32.6 emu/g, and Hc 3199 Oe was prepared by a solid phase reaction using oily CRM sludge as the source of iron. To our knowledge, preparation of nano-SrFe_12_O_19_ powder from waste by the citrate precursor method has not yet been reported.

Thus, the aim of the present paper is to report the preparation of nano-SrFe_12_O_19_ powder using oily CRM sludge as a source of iron via citrate precursor method. In addition, we also investigated the effect of annealing temperature and Fe^3+^/Sr^2+^ molar ratio in the gel on the crystal structure, morphologies, and magnetic properties of nano-SrFe_12_O_19_ powder. The results of our study show that the proposed method presents a viable alternative for recycling industrial solid waste, and the results are helpful to understand how to control the composition and magnetic properties of nano-SrFe_12_O_19_.

## 2. Materials and Methods

### 2.1. Materials

Chemically grade sulfuric acid (H_2_SO_4_, 95–98%), nitric acid (HNO_3_, 65–68%), strontium carbonate (SrCO_3_, ≥97%), sodium hydroxide (NaOH, ≥96%), hydrogen peroxide solution (H_2_O_2_, ≥97%), citric acid (C_6_H_8_O_7_·H_2_O, ≥99%), and ammonia water (NH_4_OH, 25–28%), were used in this study. The oily CRM sludge used in this study was obtained from a plant that manufactures cold rolled strip in China. The main components of oily CRM sludge are provided in Table 1 together with their content.

### 2.2. Treatment of Oily CRM Sludge

To avoid the production of toxic nitrogen oxides by direct HNO_3_ leaching, the oily CRM sludge was first leached by using 6 mol/L H_2_SO_4_ at 85 °C for 4 h under continuous agitation. The ratio of oily CRM sludge to acid was 1:5. After leaching, filtration and centrifugation were employed to separate the acid-insoluble matter and organic compounds from the leaching solution. Then, 30 wt % H_2_O_2_ was added drop wise to the leaching solution until Fe^2+^ was completely oxidized to Fe^3+^. While stirring, 5 mol/L NaOH solution, which was used as precipitant, was added to the oxidized solution until the pH reached approximately 5. As a result, ferric hydroxide (Fe(OH)_3_) precipitated. The precipitate was removed by filtration and washed several times. Finally, solutions of ferric nitrate (Fe(NO_3_)_3_) and strontium nitrate were obtained after leaching the obtained Fe(OH)_3_ precipitates and SrCO_3_ by using 8 mol/L HNO_3_, respectively. The above processes can be expressed as follows:

Fe (s) + H_2_SO_4_ (aq) → FeSO_4_ (aq) + H_2_ (g) (1)

Fe_2_O_3_ (s) + 3H_2_SO_4_ (ag) → Fe_2_(SO_4_)_3_ (ag) + 3H_2_O (L) (2)

FeO (s) + H_2_SO_4_ (ag) → FeSO_4_ (ag) + H_2_O (L) (3)

2FeSO_4_ (aq) + H_2_SO_4_ (aq) + H_2_O_2_ (L) → Fe_2_(SO_4_)_3_ (aq) + 2H_2_O (L) (4)

Fe_2_(SO_4_)_3_ (aq) + 6NaOH (aq) → 2Fe(OH)_3_ (s) + 3Na_2_SO_4_ (aq) (5)

### 2.3. Preparation of Strontium Ferrites

**Figure 1:**
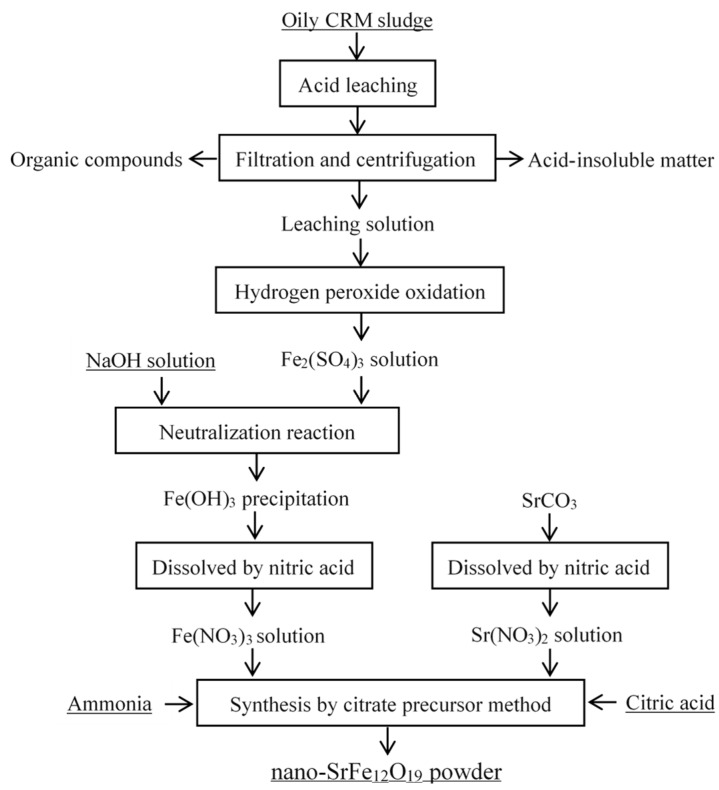
The process flow chart of SrFe_12_O_19_ powder obtained from oily CRM sludge.

Mixed solutions were prepared by varying the molar ratio of Fe^3+^/Sr^2+^ from 11.6 to 12 by proportionally mixing solutions of Fe(NO_3_)_3_ and Sr(NO_3_)_2_. Then, citric acid was added to the mixed solution until the molar ratio of citric acid to the sum of Fe^3+^ and Sr^2+^ reached 1.5. Subsequently, ammonia solution (25%) was added to the mixed solution to form a solution of pH 7. A viscous gel was obtained after magnetically stirring the solution for 4 h at 60 °C. The gel was dried at 100 °C overnight, and then burned spontaneously in air. Finally, SrFe_12_O_19_ powder was obtained after the combustion product was annealed at 400–1100 °C for 2 h in flowing air. The process flow chart of SrFe_12_O_19_ powder from oily CRM sludge is shown in Figure 1.

### 2.4. Characterization

Inductively coupled plasma (ICP, OPTIMA 7000DV, PerkinElmer) was used to analyze the chemical composition of samples. The pH values of solutions were measured by a pH/mV meter (pHS-25, Huguang, China). The morphology of the products was observed by field-emission scanning electron microscopy (FE-SEM, Zeiss Ultra 55). Fourier transform infrared (FTIR) spectroscopy (Nicolet Nexus-470, Perkin-Elmer) was used to detect the types of functional groups present in the products. Thermogravimetric and differential scanning calorimetry (TG-DSC, STA409C, Netzsch) measurements of the samples were recorded at a heating rate of 10 °C/min in air. The magnetic properties of the obtained SrFe_12_O_19_ powder were assessed using a vibrating sample magnetometer (VSM, LDJ 9600) at room temperature. The hysteresis loops were used to determine the values of Ms, Mr, and Hc. The crystalline phases present in samples were identified by X-ray diffraction (XRD, APD-10, Philips). The mean crystallite size was determined using the Scherrer formula [28]:

*d* = *Kλ*/*β*·cos*θ* (6)

where *d* is the mean crystallite size, *K* is a constant, *β* is the half width of the relevant diffraction reflection, *λ* is the X-ray wavelength, and *θ* is the diffraction angle. Moreover, the relative content of phases were calculated by the reference intensity ratio (RIR) method [29].

## 3. Results and Discussion

### 3.1. Effect of Annealing Temperature

**Figure 2:**
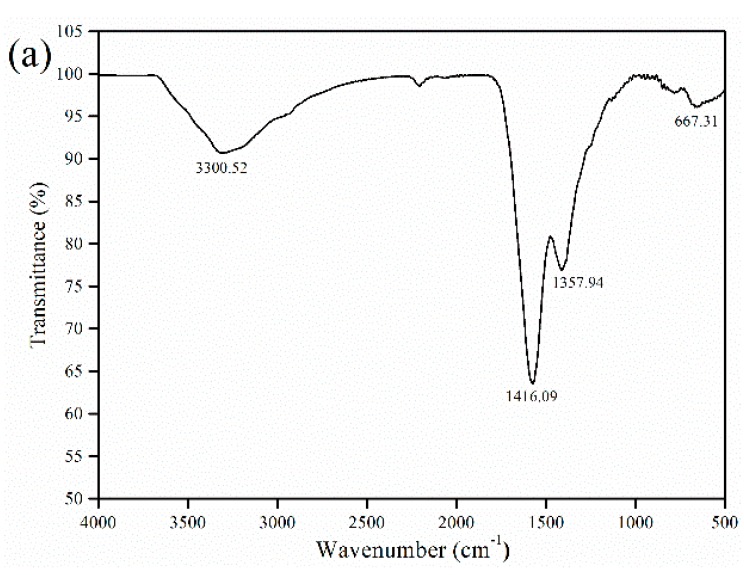

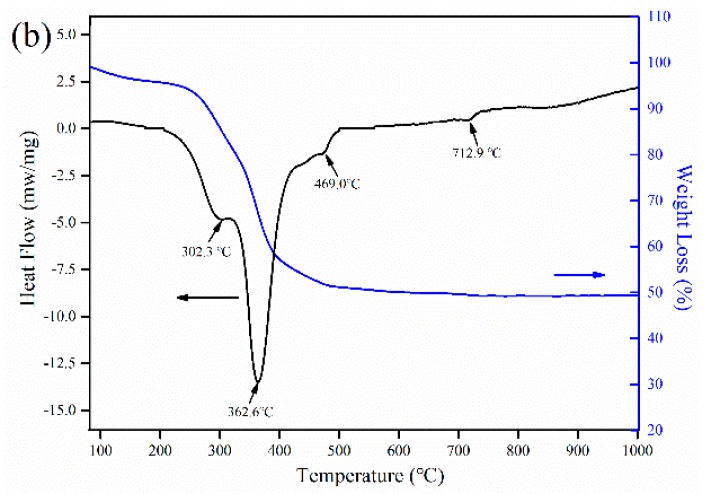
Analyses of the combustion products: (**a**) FTIR spectrum and (**b**) thermogravimetric and differential scanning calorimetry (TG-DSC) thermogram.

The effect of the annealing temperature on the formation of SrFe_12_O_19_ was investigated by fixing the Fe^3+^/Sr^2+^ molar ratio of the gel at the stoichiometric ratio of 12. After combustion of the dried gel, the resulting material was first analyzed by FTIR and TG-DSC, respectively. The FTIR peak (Figure 2a) at 3300 cm^−1^ is assigned to the vibration absorption of the O–H bond in citrate, which indicates the presence of citrate in the combustion products. The peaks at 1358 cm^−1^ and 1416 cm^−1^ are associated with the characteristic vibrational absorption band of NO_3_^−^. The broadened absorption peak near 667 cm^−1^ is the characteristic peak of γ-Fe_2_O_3_, which is associated with the Fe–O vibration. According to the TG-DSC analysis (Figure 2b), three distinct changes occur in the sample weight, that is, a small decrease below 280 °C, a significant decrease in the range of 280–470 °C, and stabilization above 470 °C.

In view of the loose and porous structure of combustion products, the weight loss of the sample below 280 °C was mainly attributed to the evaporation of adsorbed moisture. Combined with the results of FTIR analysis, the significant weight loss at 280–470 °C was mainly caused by the decomposition of residual citrate, nitrate, etc. As the temperature rose above 362.6 °C, the thermal behavior of the sample changed from endothermic to exothermic. This indicates that the exothermic effect resulting from the decomposition of NH_4_NO_3_ (shown as equation (7)) begins to play a dominant role.

2NH_4_NO_3_ → 2N_2_ (g)+ O_2_ (g) + 4H_2_O (exothermic reaction) (7)

**Figure 3:**
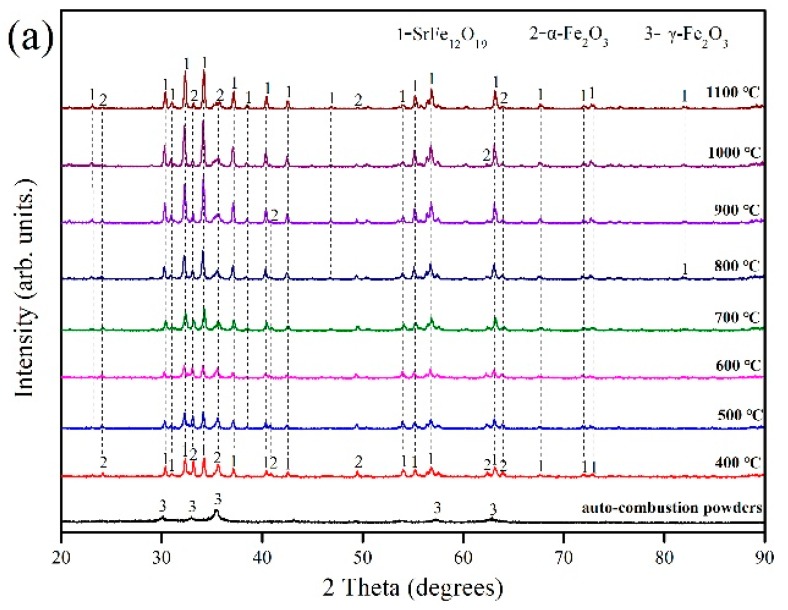

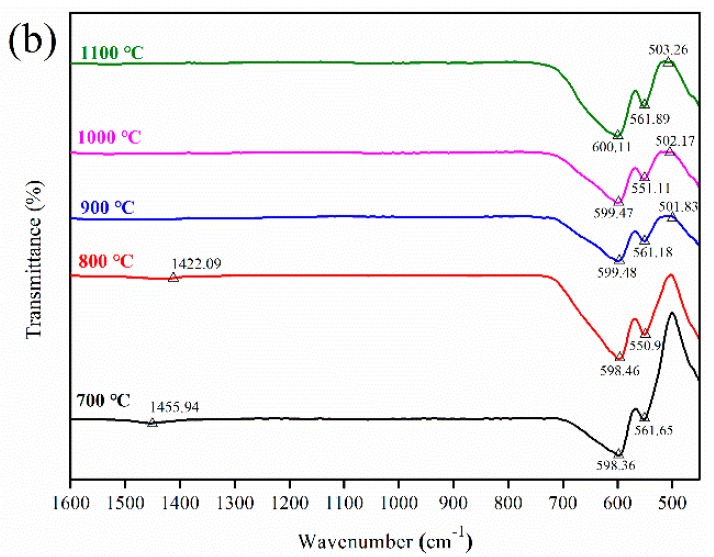
Analyses of untreated and heat-treated samples: (**a**) XRD patterns and (**b**) FTIR spectra.

To further investigate the phase changes the samples undergo during heat treatment, a series of experiments was performed by varying annealing temperature from 400 to 1100 °C. XRD patterns of untreated and heat-treated samples are shown in Figure 3a.

The main crystal phase of the untreated sample was observed to be γ-Fe_2_O_3_, whereas the main crystalline phases of the sample annealed at 400 °C are SrFe_12_O_19_ and α-Fe_2_O_3_. This indicates that the following reaction occurs during the annealing process.

γ-Fe_2_O_3_ → α-Fe_2_O_3_ (8)

The intensity and resolution of the diffraction peaks of the SrFe_12_O_19_ phase in the samples increased as the calcination temperature increased, especially above 700 °C. The increased annealing temperature significantly reduced the number and intensity of the α-Fe_2_O_3_ diffraction peaks in the sample. However, even at 1100 °C, a small amount of the α-Fe_2_O_3_ phase still existed in the sample. The samples that were obtained at various temperatures from 700 °C upward were further studied by recording their FTIR spectra (Figure 3b). The bands at 598.36 cm^−1^, 598.46 cm^−1^, 599.48 cm^−1^, 599.47 cm^−1^ and 600.11 cm^−1^ correspond to the Sr–O stretching vibration band [30]. The bands at 561.65 cm^−1^, 550.90 cm^−1^, 561.18 cm^−1^, 551.11 cm^−1^ and 561.89 cm^−1^ were attributed to the Fe–O stretching vibration by Fe–O_4_ [31]. The bands at 501.83 cm^−1^, 502.17 cm^−1^ and 503.26 cm^−1^ can be assigned to the Fe–O stretching vibrations of α-Fe_2_O_3_ [32]. This indicates the existence of SrFe_12_O_19_ and α-Fe_2_O_3_ in the samples, and is consistent with the results of the XRD analysis. Moreover, the samples obtained at 700 °C and 800 °C exhibited absorption peaks in the range 1400–1459 cm^−1^, and these peaks are associated with the characteristic vibrational absorption band of NO_3_^−^. This indicates that a certain amount of nitrate still existed in the samples below 900 °C, and that higher temperatures were helpful to remove them. Accordingly, this explains the 0.68% weight loss detected in the TG-DSC experiment.

**Figure 4:**
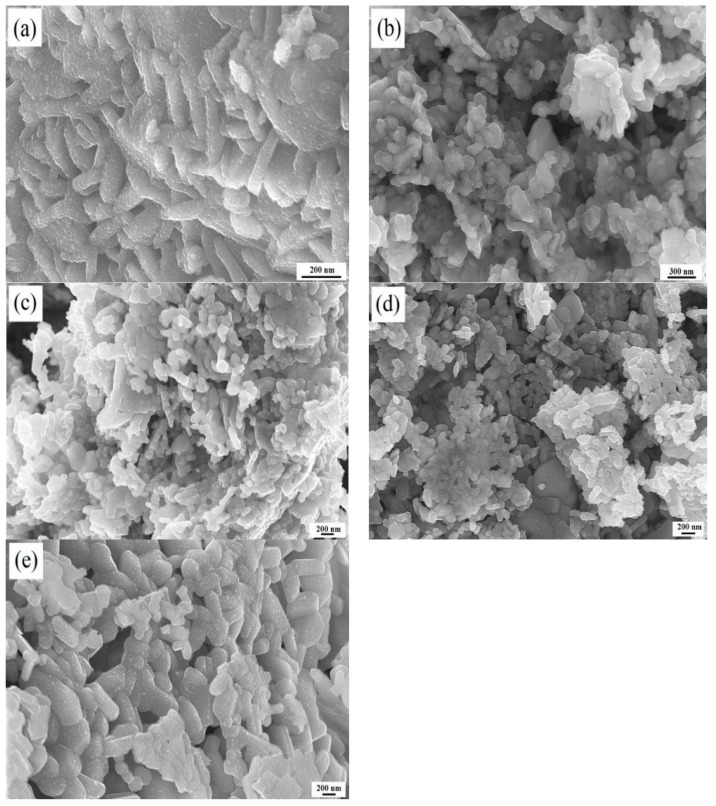
SEM images of products obtained at different annealing temperatures: (**a**) 700 °C; (**b**) 800 °C; (**c**) 900 °C; (**d**) 1000 °C; (**e**) 1100 °C.

The SEM images of the samples obtained at different annealing temperatures (Figure 4) show that the samples obtained at 700 °C and 800 °C had poor homogeneity with extensive agglomeration, indicating that the formation of SrFe_12_O_19_ was incomplete. These results are in good agreement with those of the XRD and FTIR analyses. Above 900 °C, samples were uniform with no obvious aggregation. The particle size of the sample annealed at 900 °C were approximately 200 nm. With the increase of annealing temperature from 900 to 1000 °C, the particle size of the sample increased slightly. However, at 1100 °C, the powder particles clearly experienced abnormal growth. This may be due to the growth of particle size.

**Figure 5:**
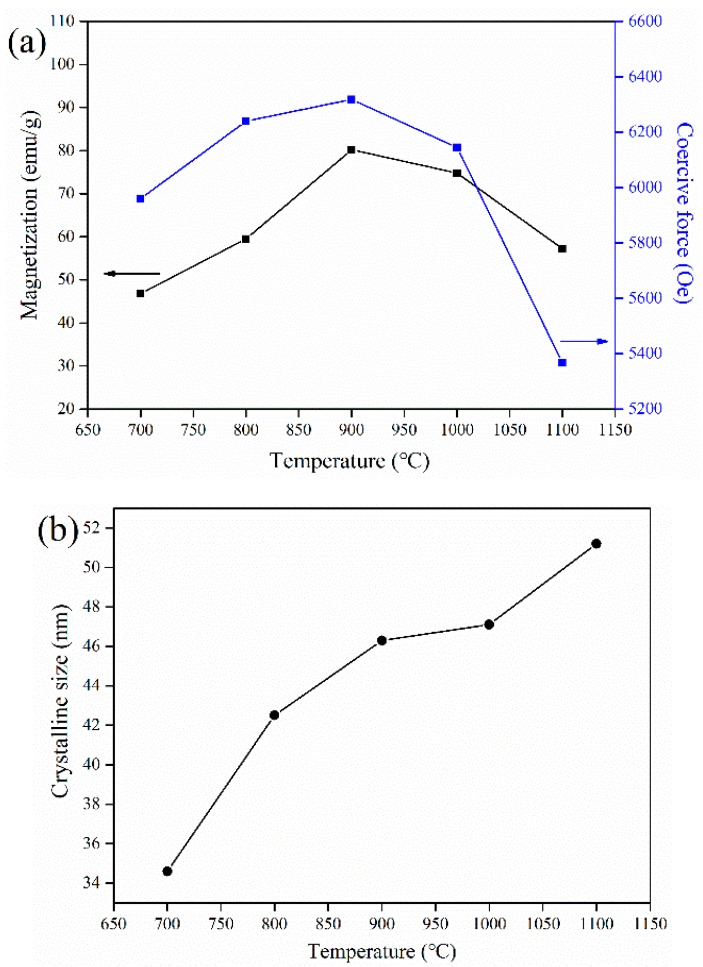
Effect of annealing temperature on the (**a**) magnetic properties and (**b**) crystalline size of the obtained SrFe_12_O_19_ powder.

The magnetic properties and crystallite size of samples as a function of temperature are summarized in Figure 5. The magnetic properties of samples increased significantly as the annealing temperature increased from 700 to 900 °C, with the highest Ms of 80.2 emu/g measured at 900 °C. Combined with the previous results, this may be ascribed to the reduction in the amount of residual nitrates, resulting in an increase in the proportion of SrFe_12_O_19_ present. Above 900 °C, the magnetic properties of the products deteriorated significantly as the annealing temperature increased. The changes in the magnetic properties can be explained by the changes in the sizes of the crystals (Figure 5b) and morphologies (Figure 4) of the samples. Excessive grain growth destroys the uniformity of samples, thus causing the deterioration of magnetic properties.

### 3.2. Effect of Fe/Sr Molar Ratio

Previous studies have shown that an appropriate Fe/Sr molar ratio is one of the decisive factors for obtaining products with a single SrFe_12_O_19_ phase [33]. In the hydrothermal synthesis of SrFe_12_O_19_, Malick et al. [34] found that products with a single SrFe_12_O_19_ phase can be obtained at a specified Fe/Sr molar ratio. According to the studies of Hessien et al. on the preparation of SrFe_12_O_19_ via the co-precipitation method [5], the pure SrFe_12_O_19_ phase can be obtained at a Fe/Sr molar ratio of 9.23 and an annealing temperature of 900 °C. Wang et al. [35] prepared SrFe_12_O_19_ powder by using the sol–gel method, and found that the pure SrFe_12_O_19_ phase can be obtained at a Fe/Sr molar ratio of 11.5 and an annealing temperature of 800 °C.

**Figure 6:**
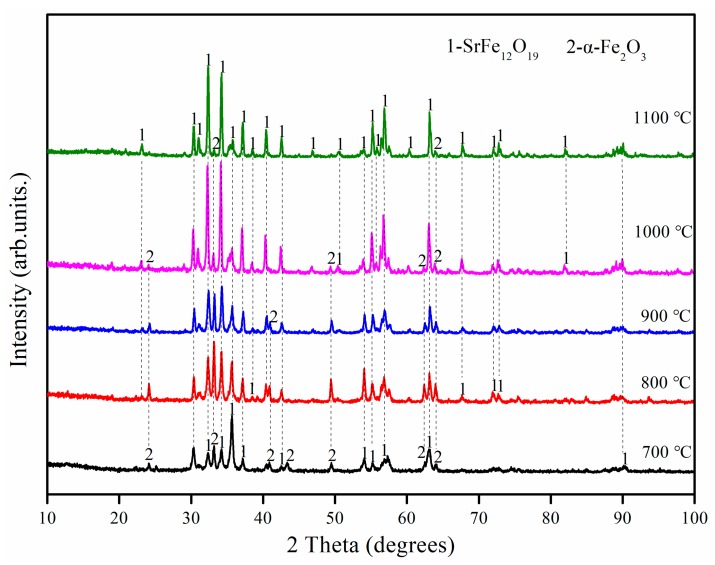
XRD patterns of SrFe_12_O_19_ with an Fe/Sr molar ratio of 11.8 and annealed at different temperatures.

Thus, to obtain products with a single SrFe_12_O_19_ phase, a series of experiments were performed by varying the Fe/Sr molar ratio from 11.6 to 11.8. Figure 6 shows the XRD patterns of products with an Fe/Sr molar ratio of 11.8 and annealed at different temperatures. The results of other analyses that were performed at the same time, including the phase content, crystalline size, and magnetic properties, are summarized in Table 2. These results indicate that the content of the α-Fe_2_O_3_ phase in the sample decreases as the annealing temperature increases. However, even at 1100 °C, it is not possible to obtain a product consisting of a single SrFe_12_O_19_ phase.

Moreover, the magnetic properties of the products listed in Table 2 increased significantly with an increase in the annealing temperature. This is mainly attributed to the increase of the SrFe_12_O_19_ phase content of the product.

**Figure 7:**
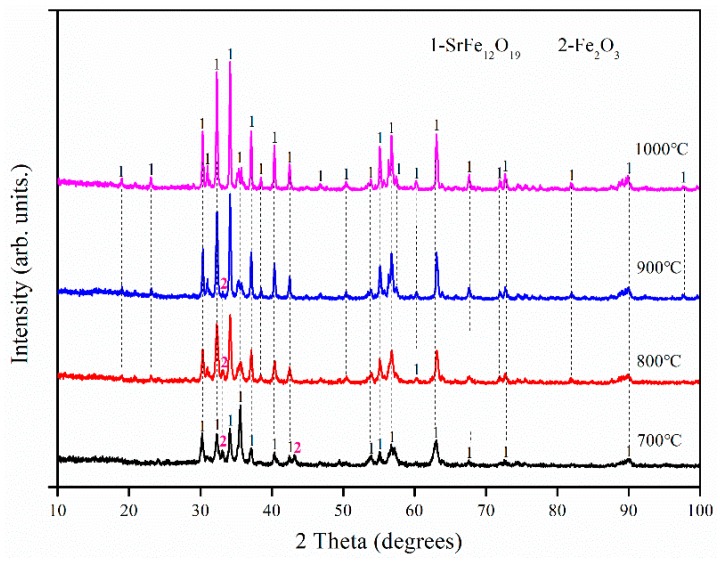
XRD patterns of SrFe_12_O_19_ with an Fe/Sr molar ratio of 11.6 by varying the annealing temperature.

Figure 7 and Table 3 present the XRD patterns of products with an Fe/Sr molar ratio of 11.4 and annealed at different temperatures. The SrFe_12_O_19_ powder samples obtained below 1000 °C contained some of the peaks associated with the α-Fe_2_O_3_ phase (7–15%). At 1000 °C, products with a well-crystallized single SrFe_12_O_19_ phase were obtained. Moreover, the results in Table 3 show that the magnetic properties of products increased by increasing the annealing temperature. This is attributed to an increase in the SrFe_12_O_19_ phase content in the product.

### 3.3. Comparison of Magnetic Properties

To summarize, two of the samples exhibited improved magnetic properties. The first is the sample with an Fe/Sr molar ratio of 12 and annealed at 900 °C, which was named SrFe_12_O_19_@900. The other is the sample with an Fe/Sr molar ratio of 11.6 and annealed at 1000 °C, which was named SrFe_11.6_O_19_@1000. These two samples were compared with those prepared from chemicals/analytical chemicals reported in the literature. The results of this comparison are presented in Table 4.

Although the content of the SrFe_12_O_19_ phase in SrFe_11.6_O_19_@1000 (100%) was higher than that in SrFe_12_O_19_@900 (97.9%), the grain size of SrFe_11.6_O_19_@1000 (74.1 nm) was significantly larger than that of SrFe_12_O_19_@900 (49.7 nm). The excessive grain growth may be the main reason why the magnetic properties of SrFe_11.6_O_19_@1000 were inferior to those of SrFe_12_O_19_@900. Moreover, Ms and Mr of SrFe_12_O_19_@900 reached 80.2 emg/g and 6318 Oe, respectively. Moreover, the comparison clearly shows that the magnetic properties of SrFe_12_O_19_@900 are competitive compared with those reported in the literature.

## 4. Conclusions

Using oily CRM sludge as an iron resource, nano-SrFe_12_O_19_ was synthesized successfully by using the citrate precursor method. The results showed that single-phase SrFe_12_O_19_ powder samples were obtained by decreasing the Fe/Sr molar ratio from the stoichiometric value of 12 to 11.6 and by increasing the annealing temperature to 1000 °C. An Fe/Sr molar ratio of 12 and annealing temperature of 900 °C produced nano-SrFe_12_O_19_ powder with a particle size of approximately 200 nm, and good magnetic properties (Ms 80.2 emu/g and Hc 6318 Oe), which are comparable to those of SrFe_12_O_19_ prepared from chemically pure materials.

## Figures and Tables

**Table 1:** Main composition of oily cold rolling mill (CRM) sludge.

Component	Content (wt %)
Fe	70.6
Ni	0.049
Mn	0.18
Cr	0.065
Si	0.058
V	0.024
Oil and moisture	18.2
Other	10.82

**Table 2:** Effect of annealing temperature on the phase content, crystalline size, and magnetic properties of the obtained SrFe_12_O_19_ samples with an Fe/Sr molar ratio of 11.8.

Annealing Temperature (°C)	Phase Content	Crystalline Size (nm)	Magnetic Properties		
			Ms (emu/g)	Mr (emu/g)	Hc (Oe)
700	73% SrFe_12_O_19_ 27% α-Fe_2_O_3_	29.6	40.8 ± 0.1	21.3 ± 0.1	854 ± 70
800	80% SrFe_12_O_19_ 20% α-Fe_2_O_3_	30.2	42.7 ± 0.1	21.8 ± 0.1	4770 ± 50
900	86% SrFe_12_O_19_ 14% α-Fe_2_O_3_	34.6	46.9 ± 0.1	24.2 ± 0.1	5260 ± 50
1000	92% SrFe_12_O_19_ 8% α-Fe_2_O_3_	44.6	59.8 ± 0.1	31.1 ± 0.1	5080 ± 40

**Table 3:** Effect of annealing temperature on the phase content, crystalline size, and magnetic properties of SrFe_12_O_19_ samples obtained with an Fe/Sr molar ratio of 11.6.

Annealing Temperature (°C)	Phase Content	Crystalline Size (nm)	Magnetic Properties		
			Ms (emu/g)	Mr (emu/g)	Hc (Oe)
700	85% SrFe_12_O_19_ 15% α-Fe_2_O_3_	45.5	45.5 ± 0.1	21.3 ± 0.1	1170.1 ± 60
800	90% SrFe_12_O_19_ 10% α-Fe_2_O_3_	50.1	50.1 ± 0.1	26.6 ± 0.1	5737.9 ± 30
900	93% SrFe_12_O_19_ 7% α-Fe_2_O_3_	58.1	58.1 ± 0.1	31.1 ± 0.1	6437.8 ± 20
1000	100% SrFe_12_O_19_	74.2	67.5 ± 0.1	36.1 ± 0.1	6176.0 ± 20

**Table 4:** Performance comparison between the samples obtained in this study and those reported in the literature.

Sample	Synthetic Method	Magnetic Properties		
		Ms (emu/g)	Mr (emu/g)	Hc (Oe)
SrFe_12_O_19_@900	CPM	80.2	39.8	6318
SrFe_11.6_O_19_@1000	CPM	67.5	36.1	6176
SrFe_12_O_19_ powder [20]	MA-SGM	54.8	29.5	5261
Sr_0.9_La_0.1_Fe_11.9_Co_0.1_O_19_ powder [20]	SGM	73	36	7700
Sr_0.85_Nd_0.15_Fe_12_O_19_ powder [21]	CPM	63	35.15	6885
SrFe_12_O_19_ nanoribbons [22]	SAE	67.9	37.3	7310
SrFe_12_O_19_ powder [23]	SGM	59.3	34.9	6725

CPM = citrate precursor method; SGM = sol–gel method; MA-SGM = microwave-assisted sol–gel method; SAE = solution assisted electrospinning.
